# Swin Transformer-Based Edge Guidance Network for RGB-D Salient Object Detection

**DOI:** 10.3390/s23218802

**Published:** 2023-10-29

**Authors:** Shuaihui Wang, Fengyi Jiang, Boqian Xu

**Affiliations:** Changchun Institute of Optics, Fine Mechanics and Physics, Chinese Academy of Sciences, Changchun 130033, China; wangshuaihui@ciomp.ac.cn (S.W.); jiangfengyi@ciomp.ac.cn (F.J.)

**Keywords:** RGB-D salient object detection, edge guidance, transformer, cross-modal interaction

## Abstract

Salient object detection (SOD), which is used to identify the most distinctive object in a given scene, plays an important role in computer vision tasks. Most existing RGB-D SOD methods employ a CNN-based network as the backbone to extract features from RGB and depth images; however, the inherent locality of a CNN-based network limits the performance of CNN-based methods. To tackle this issue, we propose a novel Swin Transformer-based edge guidance network (SwinEGNet) for RGB-D SOD in which the Swin Transformer is employed as a powerful feature extractor to capture the global context. An edge-guided cross-modal interaction module is proposed to effectively enhance and fuse features. In particular, we employed the Swin Transformer as the backbone to extract features from RGB images and depth maps. Then, we introduced the edge extraction module (EEM) to extract edge features and the depth enhancement module (DEM) to enhance depth features. Additionally, a cross-modal interaction module (CIM) was used to integrate cross-modal features from global and local contexts. Finally, we employed a cascaded decoder to refine the prediction map in a coarse-to-fine manner. Extensive experiments demonstrated that our SwinEGNet achieved the best performance on the LFSD, NLPR, DES, and NJU2K datasets and achieved comparable performance on the STEREO dataset compared to 14 state-of-the-art methods. Our model achieved better performance compared to SwinNet, with 88.4% parameters and 77.2% FLOPs. Our code will be publicly available.

## 1. Introduction

Salient object detection (SOD) is an important preprocessing method in computer vision tasks, with applications in video detection and segmentation [1], semantic segmentation [2], object tracking [3], etc.

CNN-based models for RGB SOD have yielded great performance in localizing salient objects [4,5,6,7,8]. However, it is still difficult to localize the salient object accurately in scenes such as those with low contrast or objects with a cluttered background. CNN-based RGB-D SOD models, which employ features from RGB images and depth maps, have attracted growing interest and presented promising performance [9,10,11,12,13,14,15,16,17,18,19,20,21,22,23]. However, some issues still limit the performance of existing CNN-based RGB-D SOD models.

The first issue is that CNN-based models cannot effectively capture long-range dependencies. Long-range semantic information plays an important role in identifying and locating salient objects [24]. Due to the intrinsic locality of convolution operations, CNN-based models cannot effectively extract global context information. In addition, the empirical receptive field of CNN is much smaller than the theoretical receptive field, especially on high-level layers [25].

The second issue is that depth maps are often noisy. The performance of RGB-D SOD models relies on reliable RGB images and depth maps. Misleading information in depth maps degrades the performance of RGB-D SOD models.

Global context information helps reduce errors created via poor depth maps. Transformers can extract features and model long-range dependencies, and Transformer-based methods have achieved outstanding performance in various computer vision tasks [26,27,28,29]. However, Transformers are less effective in capturing local features. The Swin Transformer [29], combining the advantages of Transformers and CNN, has been shown to have a powerful feature extraction ability. Considering the above challenges, the Swin Transformer is suitable as a feature extractor for RGB-D SOD tasks.

Swin Transformer-based models are relatively weak in their ability to model local context information. Therefore, Swin Transformer-based models should pay more attention to local feature information.

Based on the investigation above, we propose a novel Swin Transformer-based edge guidance network (SwinEGNet) that enhances feature locality to boost the performance of RGB-D SOD. We employed the Swin Transformer as the backbone to extract features from RGB images and depth maps for capturing long-range dependencies. We introduced a depth enhancement module (DEM) and a cross-modal interaction module to enhance local features. Unlike other methods, we employed edge clues to enhance depth features rather than edge clues as decoder guidance to directly refine the final prediction map. We designed the edge extraction module (EEM) to extract edge information and the depth enhancement module (DEM) to enhance depth features. Furthermore, we used a cross-modal interaction module to effectively integrate information from global and local contexts. To effectively explore the features of each layer, we employed a cascaded decoder to progressively refine our saliency maps.

**Figure 1:**
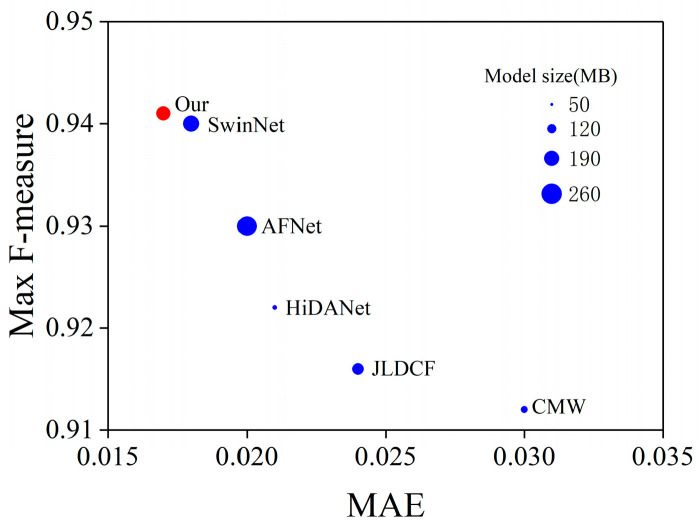
Max F-measure, MAE, and model size of different methods on the NLPR dataset. Our model achieves better performance with a smaller model size.

Our main contributions are summarized as follows:

- A novel edge extraction module (EEM) is proposed, which generates edge features from the depth features.
- A newly designed edge-guided cross-modal interaction was employed to effectively integrate cross-modal features, where the depth enhancement module was employed to enhance the depth feature and the cross-modal interaction module was employed to encourage cross-modal interaction from global and local aspects.
- A novel Swin Transformer-based edge guidance network (SwinEGNet) for RGB-D SOD is proposed. The proposed SwinEGNet was evaluated with four evaluation metrics and compared to 14 state-of-the-art (SOTA) RGB-D SOD methods on six public datasets. Our model achieved better performance with less parameters and FLOPs than SwinNet, as shown in Figure 1. In addition, a comprehensive ablation experiment was also conducted to verify the effectiveness of the proposed modules. The experiment results showed the outstanding performance of our proposed method.

The remainder of this paper is structured as follows: The current status of RGB-D salient object detection is presented in Section 2. The overall architecture, detailed structure, and loss function of the proposed network are outlined in Section 3. The results of our experiments are provided in Section 4. Finally, our conclusions are presented in Section 5.

## 2. Related Work

CNN-based RGB-D salient object detection: Benefitting from the development of deep learning and depth sensors, many CNN-based RGB-D SOD methods have recently been proposed. Compared to RGB SOD methods, RGB-D SOD models employ depth clues as complementary information and have shown outstanding performance in salient object detection. Most RGB-D SOD models adopt CNN-based networks to extract features and focus on cross-modal fusion strategies to improve salient object detection performance. Various frameworks and fusion strategies have been proposed to effectively merge cross-modal cross-scale features [14,17,21,22,23,30,31]. Zhang et al. [30] designed an asymmetric two-stream network, where a flow ladder module is introduced to the RGB stream to capture global context information and DepthNet for the depth stream. Zhang et al. [17] proposed a multistage cascaded learning framework for RGB-D saliency detection, which minimizes the mutual information between RGB images and depth maps to model complementary information. Chen et al. [22] designed a triplet encoder network that processes RGB, depth, and fused features separately to suppress the background noise in the depth map and sharpen the boundaries of high-level features. Li et al. [14] designed a hierarchical alternate interaction module that progressively and hierarchically integrates local and global contexts. Wu et al. [21] proposed layer-wise, trident spatial, and attention mechanisms to fuse robust RGB and depth features against low-quality depths. Wu et al. [23] employed a granularity-based attention module to leverage the details of salient objects and introduced a dual-attention module to fuse the cross-modal cross-scale features in a coarse-to-fine manner.

To address the insufficiency of obtaining global semantic information of CNN-based networks, Liu et al. [7] proposed using a receptive field block to enhance feature discriminability and robustness by enlarging the receptive field. Dilated convolutions can enlarge the receptive field of CNN without loss of resolution. As a result, Yu et al. [32] presented modules based on dilated convolutions to aggregate multiscale information. Liu et al. [8] designed a global guidance module for RGB SOD that utilizes the revised pyramid pooling module to capture global semantic information.

Transformer-based RGB-D salient object detection: The Transformer was first employed for machine translation and gradually introduced in computer vision tasks. Dosovitskiy et al. [26] proposed the first Vision Transformer (ViT), Wang et al. [28] proposed a progressive shrinking pyramid Transformer (PVT), and Liu et al. [29] designed the Swin Transformer. Subsequently, researchers employed the Transformer as the backbone network to improve the detection performance of RGB-D SOD. Liu et al. [33] developed a unified model based on ViT for both RGB and RGB-D SOD. Zeng et al. [34] employed the Swin Transformer as the encoding backbone to extract features from RGB images and depth maps. Liu et al. [35] employed PVT as a powerful feature extractor to extract global context information and designed a lightweight CNN-based backbone to extract spatial structure information in depth maps. Pang et al. [36] proposed using a novel top-down information propagation path based on the Transformer to capture important global clues to promote cross-modal feature fusion. Liu et al. [37] proposed using a cross-modal fusion network based on SwinNet for RGB-D and RGB-T SOD. Roy et al. [38] employed the Swin Transformer as the encoder block to detect multiscale objects.

## 3. Methodologies

In this section, we present the proposed Swin Transformer-based edge guidance network (SwinEGNet). We provide an overview of our method and describe its main components in detail, including the feature encoder, edge extraction module, edge-guided cross-modal interaction module, cascaded decoder, and loss function.

### 3.1. The Overall Architecture

**Figure 2:**
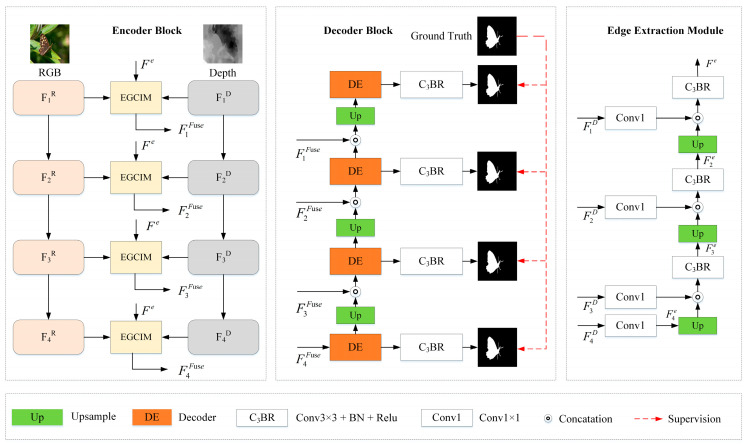
An overview of the proposed SwinEGNet. It consists of a feature encoder, an edge extraction module (EEM), an edge-guided cross-modal interaction module (EGCIM), and a cascaded decoder.

As illustrated in Figure 2, we present a Swin Transformer-based edge guidance network (SwinEGNet). Inspired by [37], we employed edge clues to guide salient object detection. However, unlike [37], edge clues were incorporated into cross-modal interaction blocks to enhance depth features rather than being employed as decoder guidance to refine the final prediction map. The proposed SwinEGNet adopts the encoder–decoder structure. As shown in Figure 2, SwinEGNet consists of a feature encoder, edge extraction module (EEM), edge-guided cross-modal interaction module (EGCIM), and cascaded decoder. Firstly, RGB images and depth maps are fed into two independent Swin Transformers for feature extraction, and an EEM is proposed to extract edge features. Then, these features are fed into EGCIM for depth feature enhancement and feature fusion, where the depth enhancement module (DEM) is responsible for depth feature enhancement and the cross-modal interaction module (CIM) is responsible for feature fusion. Finally, the fused features are fed into the decoder block for saliency maps. The cascaded decoder was employed to effectively explore the features of the four layers and progressively refine the saliency maps.

### 3.2. Feature Encoder

In contrast to other Transformers, the Swin Transformer computes multihead self-attention within a local window instead of the whole input to model locality relationships. Furthermore, it employs a shifted window operation to model long-range dependence across windows. Therefore, the Swin Transformer is suitable for feature extraction because it incorporates the merits of the Transformer and CNN. Considering the performance and computational complexity, we adopted the Swin-B Transformer as the backbone to extract features from RGB images and depth maps, which accept an input size of 384 × 384.

RGB images and depth maps are fed into two independent Swin Transformers for feature extraction. Considering the first layer contains redundant noisy information, the extracted features of the last four layers are employed for feature fusion. The features can be expressed as follows:

(1) $$F_{i}^{R}=trans(I_{R}),i=1,2,3,4$$

(2) $$F_{i}^{D}=trans(I_{D}),i=1,2,3,4$$

where $F_{i}^{R}$ denotes the RGB feature; $F_{i}^{D}$ denotes the depth feature, trans(⋅) denotes the Transformer; and $I_{R}$ and $I_{D}$ denote the input RGB image and depth image, respectively.

### 3.3. Edge Extraction Module

**Figure 3:**
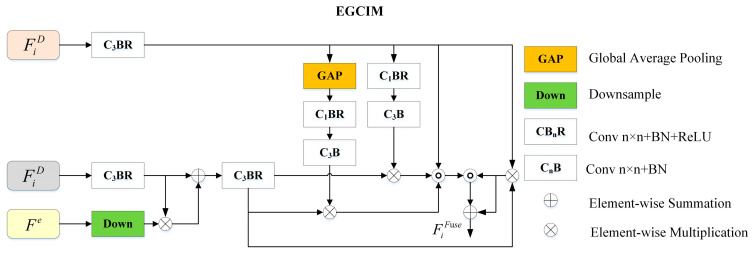
Details of the proposed edge-guided cross-modal interaction module (EGCIM).

To extract edge features, we propose an edge extraction module (EEM). The extracted edge features are fed into EGCIM to enhance the depth feature. The details of the proposed EEM are illustrated in Figure 3.

Shallow layers contain low-level information such as structure clues, while deep layers contain global semantic information. They are all helpful in extracting edge information. In contrast to other methods that employ parts of the depth features for edge prediction, we employed all depth features for edge extraction, and the edge features were progressively refined in a coarse-to-fine manner.

In particular, the depth features $F_{i}^{D}(i=1,2,3,4)$ are fed into a 1 × 1 convolutional layer for channel reduction. Then, features $F_{i}^{D}(i=2,3,4)$ perform the upsample operation to generate the same size features as $F_{i+1}^{D}$. The edge feature $F_{4}^{e}$ can be expressed as follows:

(3) $$F_{4}^{e}=Up\left(Conv_{1}(F_{4}^{D})\right)$$

where $Up\left(\cdot \right)$ denotes the upsample operation.

Next, the edge feature performs a concatenation operation and a 3 × 3 convolutional layer with a BatchNorm and a ReLU function to generate the edge feature $F_{i-1}^{e}$, which can be expressed as follows:

(4) $$F_{i}^{e}=C_{3}BR\left(Cat\left(Conv_{1}(F_{i}^{D}),F_{i+1}^{e}\right)\right),i=1,2,3$$

where $C_{3}BR\left(\cdot \right)$ denotes a 3 × 3 convolutional layer with a BatchNorm and a ReLU function, and $Cat\left(\cdot \right)$ denotes concatenation operation. The edge feature $F_{1}^{e}$ is the final edge feature $F^{e}$. The final edge feature $F^{e}$ will be fed into EGCIM for depth enhancement.

### 3.4. Edge-Guided Cross-modal Interaction Module

To enhance depth features and encourage cross-modal feature interaction, we designed an edge-guided cross-modal interaction module (EGCIM) to integrate features from both modalities, including a depth enhancement module (DEM) and a cross-modal interaction module (CIM).

Depth enhancement module: Though Transformer-based methods sufficiently capture global context information, they are relatively weak at capturing local context information compared to CNN-based methods. Therefore, it is necessary to utilize local clues like edge information to enhance the depth features. We designed a depth enhancement module (DEM) to enhance the depth features, which introduces edge information extracted from the depth features to these features for depth enhancement. The detailed structure of DEM is shown in Figure 3.

The depth features $F_{i}^{D}$ and edge features $F^{e}$ at a certain hierarchy i=1,2,3,4, $F_{i}^{D}$ performs the convolution operation with a BatchNorm and a ReLU function for channel reduction, and $F^{e}$ performs the downsample operation to gain the same size as $F_{i}^{D}$. Then, the depth features $F_{i}^{D}$ and edge features of the same size are fused using multiplication and addition operations. The enhanced depth features can be expressed as follows:

(5) $$F_{i}^{DE}=C_{3}BR\left(C_{3}BR(F_{i}^{D})+C_{3}BR(F_{i}^{D})\times Down(F^{e})\right)$$

where + denotes the addition operation, and Down(⋅) denotes the downsample operation. The enhanced depth features $F_{i}^{DE}$ will be fed into CIM for feature fusion.

Cross-modal interaction module: We used a cross-modal interaction module (CIM) to effectively combine RGB and depth modalities. The CIM contains a global attention branch and a local attention branch to enhance globality and locality. In addition, a residual connection is adopted to combine the fused features with RGB features for the preservation of the RGB images’ original information. The local information of the depth features enhances the RGB features to sharpen the details of salient objects, and the global context information of the depth features enhances the RGB features to locate the salient object.

As shown in Figure 3, the RGB features are fed into a 3 × 3 convolutional layer with a BatchNorm and a ReLU activation function for channel reduction. There are three branches for feature fusion: the first branch employs global average pooling (GAP) to capture global context information, the second branch employs 1 × 1 convolution to obtain local information, and the third branch aims to keep the original information of RGB features. Then, we carry out multiplication, concatenation, and addition operations for fusion. The fused features can be expressed as follows:

(6) $$F_{i}^{Fuse}=C_{3}BR\left(C_{3}BR\left(Cat\left(C_{3}BR(F_{i}^{R}),F_{i}^{g},F_{i}^{l},F_{i}^{o}\right)\right)+F_{i}^{o}\right)$$

(7) $$F_{i}^{Fuse}=C_{3}BR(F_{i}^{R})\times F_{i}^{DE}$$

(8) $$F_{i}^{g}=C_{3}BR(F_{i}^{R})\times C_{1}B\left(C_{1}BR\left(GAP(F_{i}^{DE})\right)\right)$$

(9) $$F_{i}^{l}=C_{3}BR(F_{i}^{R})\times C_{1}B\left(C_{1}BR(F_{i}^{DE})\right)$$

where $GAP\left(\cdot \right)$ represents the global average pooling operation, $C_{1}B$ represents a convolution operation with a BatchNorm function, $C_{1}BR$ represents a convolution operation with a BatchNorm function and a ReLU function, and $F_{i}^{Fuse}$ represents the fused features.

### 3.5. Cascaded Decoder

The cascaded encoder can effectively leverage the multilevel features and eliminate the noise in low-level features, which improves the accuracy of salient maps. Moreover, deep-layer supervision performs better than single supervision [13]. Therefore, we employed a cascaded decoder for the final prediction map, as shown in Figure 3. The decoder has four decoding levels corresponding to the four-level cross-modal feature interaction. Consequently, the prediction map is refined progressively. Each decoder contains two 3×3 convolution layers with a BatchNorm and a ReLU function, a dropout layer, and an upsample layer. The initial prediction map $S_{4}$ is fed into the decoder and concatenates with the previous prediction map $S_{n-1}$ for refinement. The prediction features $S_{i}$ can be donated as follows:

(10) $$S_{i}=\left\{\begin{array}{l} C_{3}BR\left(Up(S_{i+1}),S_{i}\right),i=1,2,3\\ C_{3}BR(F_{i}^{Fuse}),i=4 \end{array}\right.$$

where $D\left(\cdot \right)$ represents the decoder operation, $S_{n}$ represents the prediction map, and $Up\left(\cdot \right)$ represents the upsample operation. Next, features $S_{i}$ perform convolution operations to obtain the prediction map, and $S_{1}$ is the final prediction map.

### 3.6. Loss Function

Detection loss is composed of the weighted binary cross-entropy (BCE) loss $L_{BCE}^{\omega }$ and the weighted intersection-over-union (IOU) loss $L_{IoU}^{\omega }$ [39], which has been invalidated in salient object detection. The detection loss can be formulated as follows:

(11) $$L_{d}=L_{BCE}^{\omega }+L_{IoU}^{\omega }$$

$L_{IoU}^{\omega }$ and $L_{BCE}^{\omega }$ pay more attention to the structure of SOD and the hard pixels to highlight the importance of the hard pixel. As illustrated in Figure 2, four-level supervisions are applied to supervise the four side-output maps. Each map $S_{i}$ is upsampled to the same size as the ground truth map. Thus, the total loss function L can be expressed as follows:

(12) $$L=\sum_{i=1}^{4}(L_{d}^{i}(S_{i},G)$$

## 4. Experiments

### 4.1. Datasets and Evaluation Metrics

Datasets: We evaluated the proposed method on six widely used benchmark datasets: STEREO (1000 image pairs) [40], NJU2K (2003 image pairs) [41], NLPR (1000 image pairs) [42], LFSD (100 image pairs) [43], SIP (929 image pairs) [44], and DES (135 image pairs) [45]. For a fair comparison, our training settings were the same as the existing works [12], which consisted of 1485 samples from the NJU2K dataset and 700 samples from the NLPR dataset. The remaining images from NLPR, DES, and NJU2K, and the whole SIP, STEREO, and LFSD were used for testing.

Evaluation metrics: We adopted four widely used evaluation metrics for quantitative evaluation, including S-measure ($S_{\alpha }$, α=0.5) [46], maximum F-measure ($F_{m}$) [47], maximum E-measure ($E_{m}$) [48], and mean absolute error (MAE, M) [49]. S-measure evaluates the structural similarity between the saliency map and ground truth, which is defined as follows:

(13) $$S=\alpha S_{o}+(1-\alpha )S_{r}$$

where α is a trade-off parameter set to 0.5, $S_{o}$ represents the object perception, and $S_{r}$ represents the regional perception. F-measure focuses on region-based similarity that considers precision and recall, which is defined as follows:

(14) $$F_{\beta }=\left(1+\beta^{2}\right)\frac{P\times R}{\beta^{2}\times P+R}$$

where P denotes precision, R denotes recall, and $\beta^{2}$ is a trade-off parameter set to 0.3. We used the maximum F-measure as the evaluation metric. MAE assesses the average absolute error at the pixel level, which is defined as follows:

(15) $$MAE=\frac{1}{W\times H}\sum_{i=1}^{W}\sum_{j=1}^{H}\left|S\left(i,j\right)-G\left(i,j\right)\right|$$

where W and H represent the width and height of the image, respectively. S represents the saliency maps, and G represents the ground truth. E-measure is employed to capture image-level statistics and local pixel matching, which is defined as follows:

(16) $$E_{m}=\frac{1}{W\times H}\sum_{i=1}^{W}\sum_{j=1}^{H}\phi_{FM}\left(i,j\right)$$

where $\phi_{FM}$ represents the enhanced alignment matrix. For a fair comparison, we used the evaluation tools provided by [15].

### 4.2. Implementation Details

We implemented our model on PyTorch with one NVIDIA A4000 GPU. The Swin Transformer that has been pretrained on ImageNet was employed as our backbone network. The parameters of the Swin-B model were initialized with the pretrained parameters, and the remaining parameters were initialized with PyTorch default settings. The Adam optimizer was employed to train the proposed model with a batch size of 5, a momentum of 0.9, and a weight decay of 0.1. The initial learning rate was 1 × 10^−4^, which was then divided by 10 for every 60 epochs. All images were resized to 384 × 384 for training and testing. The single-channel depth image was replicated to a three-channel image, which was the same as the RGB image. Data augment strategies, including random flipping, rotating, and border clipping, were employed to augment the training data. The model was trained for 120 epochs.

### 4.3. Comparison with SOTAs

Quantitative comparison: We compared the proposed network with 14 SOTA CNN-based methods and Transformer-based methods, which were CMW [13], JLDCF [50], HINet [51], DSA2F [20], CFIDNet [52], C^2^DFNet [53], SPSNet [19], AFNet [22], HiDANet [23], MTFormer [54], VST [43], TANet [35], and SwinNet [37]. The compared saliency maps were directly provided by the authors or generated via their released codes. The quantitative comparison under four evaluation metrics on six datasets is shown in Table 1. As shown in Table 1, our SwinEGNet performed the best on LFSD, NLPR, and DES datasets and competitively performed on NJU2K, STEREO, and SIP datasets. In particular, SwinEGNet performed outstandingly on the LFSD dataset, which is considered a challenging dataset. Compared to the second model DSA2F, the improvements of S-measure, F-measure, E-measure, and MAE were about 0.011, 0.006, 0.005, and 0.002, respectively. On the NJU2K dataset, the performance of our method was comparable with SwinNet. On the STEREO dataset, our method performed the best in $E_{m}$.

**Figure 4:**
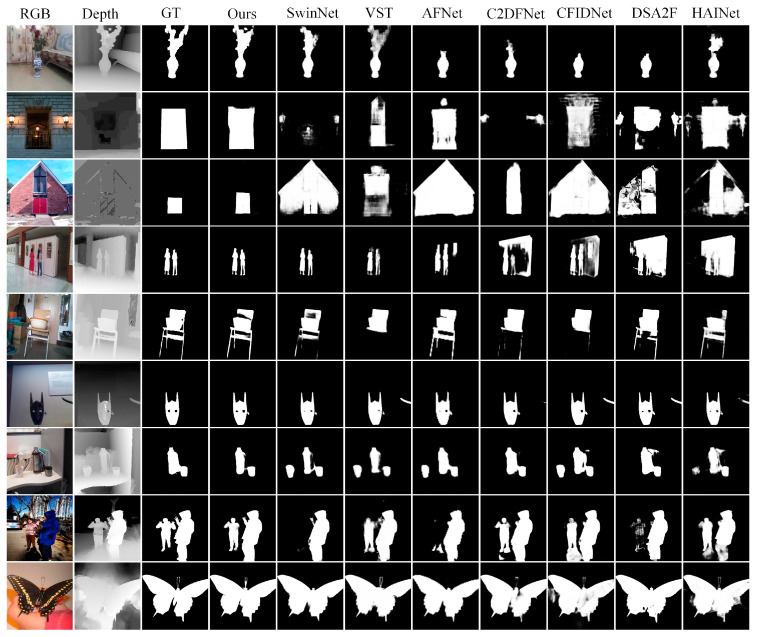
Visual comparison of our method and seven SOTAS, including CMW, DSA2F, CFIDNet, C^2^DFNet, AFNet, VST, and SwinNet.

Qualitative comparison: We qualitatively compared seven representative methods on challenging scenes. The first scene had a similar foreground and background (first row), the second scene had poor depth map (second row and third row), the third scene had a complex background (fourth row and fifth row), the fourth scene had a small object (sixth row), the fifth scene had multiple objects (seventh row and eighth row), and the sixth scene had a fine structure (ninth row). As shown in Figure 4, our method obtained the best detection results. For the first scene, the foreground and background of the RGB image were similar, but the depth map provided correct information. Our method located salient objects better than other methods thanks to the power of EEM and EGCIM. For the second scene, though the depth map provided incorrect information, our method successfully located salient objects by eliminating misleading information of the poor depth map. For the fourth scene, our method fused the RGB feature and depth feature the best. For the fifth scene, our method not only located the salient objects but also maintained the sharp boundaries. These all indicate the effectiveness of our model.

### 4.4. Ablation Study

We conducted comprehensive ablation studies on LFSD and STEREO datasets to evaluate the effectiveness of the proposed modules in our proposed model.

**Figure 5:**
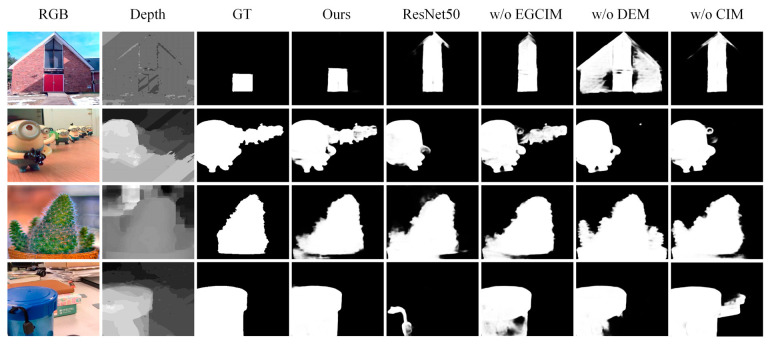
Visual comparison of the ablation study.

Effectiveness of Swin Transformer backbone: We replaced the feature encoder with ResNet50 to verify the effectiveness of the Swin Transformer backbone. As shown in Table 2, the Transformer-based model showed better performance in all the evaluation benchmarks and metrics, especially on the LFSD dataset. We show the visual comparison of ResNet50 and Swin Transformer in Figure 5. The ResNet50 was inferior to the Swin Transformer. This validates the effectiveness of the Swin Transformer backbone for the RGB-D SOD.

Effectiveness of EGCIM: To explore the effectiveness of EGCIM, we replaced EGCIM with a multiplication operation. In Table 2, we quantitatively demonstrate the contribution of the EGCIM. The performance of our model degraded without the help of EGCIM. This validates the effectiveness of the edge-guided cross-modal interaction module.

Effectiveness of DEM in EGCIM: To verify the effectiveness of DEM in EGCIM, we removed DEM from our full model. In Table 2, we quantitatively demonstrate the contribution of DEM. As shown in Table 2, the depth enhancement module improved the performance of the proposed model, especially on the LFSD dataset. The MAE, S-measure, F-measure, and E-measure are improved by about 0.012, 0.018, 0.013, and 0.009 in the LFSD dataset, respectively.

Effectiveness of CIM in EGCIM: We replaced CIM with a multiplication operation to verify the effectiveness of CIM in EGCIM. In Table 2, we quantitatively demonstrate the contribution of CIM. As shown in Table 2, the performance degradation caused by removing CIM supports our claim that the cross-modal interaction module can effectively fuse the RGB and depth features.

### 4.5. Complexity Analysis

We conducted a complexity comparison with the other five models on the number of parameters and FLOPs, as shown in Table 3. The performance of the CNN-based models was relatively poor compared to the Transformer-based models. Our model performed better with fewer parameters and lower computational costs compared to SwinNet. The parameters and FLOPs of our model were 175.6 M and 96 G, respectively. Our model achieved comparable performance to SwinNet, yielding 88.4% parameters and 77.2% FLOPs.

### 4.6. Failure Cases

**Figure 6:**
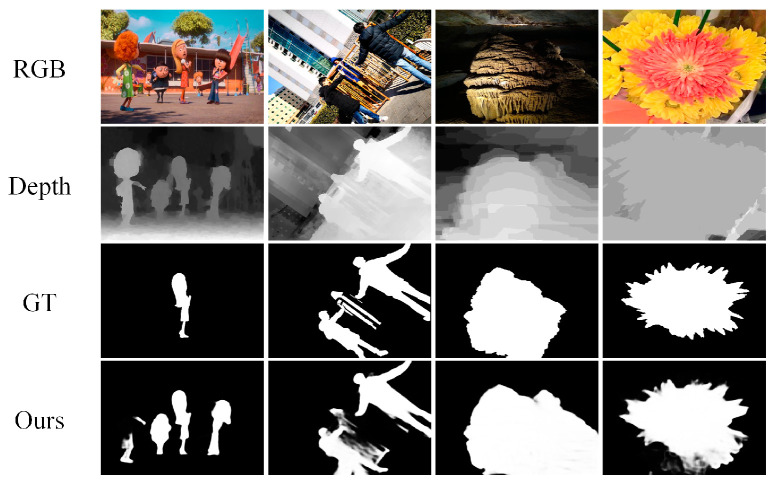
Visualization of failure cases in challenging scenes.

We show some failure cases on the challenging scenes in Figure 6: the first scene with multiple objects (first row and second column), and the second scene with poor depth map (third row and fourth row). As shown in the first scene, our model could not accurately locate multiple objects with complex backgrounds. Global feature relations are important for locating multiple salient objects. Multihead self-attention within a local window enhanced the locality, but it also limited the long-range model ability of the Swin Transformer. The second scene indicates that our model could not locate salient objects well in some scenes with poor depth maps. In addition to the low quality of depth maps, there were misalignments between RGB images and depth maps at the pixel level. It is difficult to effectively fuse features for direct pixel-wise fusion. We will conduct further research in the future.

## 5. Conclusions

In this paper, we propose a novel Swin Transformer-based edge guidance network for RGB-D SOD. We employed the Swin Transformer as the backbone to extract features from RGB images and depth maps for capturing the long-range dependencies. Additionally, we proposed using the edge extraction module (EEM), the depth enhancement module, and the cross-modal interaction module (CIM) to enhance the local features. The EEM extracts edge features from the depth features, and the DEM employs edge information to enhance the depth features. The CIM effectively fuses RGB features and depth features from global and local contexts. With all these modules working together, our SwinEGNet model can accurately localize salient objects in various complex scenarios with sharp boundaries. Countless comparison studies and ablation experiments demonstrated that the proposed SwinEGNet showed outstanding performance on six widely used RGB-D SOD benchmark datasets. As an independent module, EEM can be applied to related tasks. In the future, we will extend our model to RGB-T salient object detection.

## Figures and Tables

**Table 1:** Quantitative comparison of SOTA methods under four evaluation metrics: S-measure ($S_{a}$), max F-measure ($F_{m}$), max E-measure ($E_{m}$), and MAE (M). ↑ denotes that higher is better, and ↓ denotes that lower is better. The best two results are shown in red and green fonts, respectively.

	Metric	CMW	JLDCF	HINet	HAINet	DSA2F	CFIDNet	C^2^DFNet	SPSNet	AFNet	HiDANet	MTFormer	VST	TANet	SwinNet	Our
LFSD	*S_m_*↑	0.876	0.854	0.852	0.854	0.882	0.869	0.863	-	0.89	-	0.872	0.89	0.875	0.886	0.893
	*F_m_*↑	0.899	0.862	0.872	0.877	0.903	0.883	0.89	-	0.9	-	0.879	0.903	0.892	0.903	0.909
	*E_m_*↑	0.901	0.893	0.88	0.882	0.920	0.897	0.899	-	0.917	-	0.911	0.918	-	0.914	0.925
	*M*↓	0.067	0.078	0.076	0.08	0.054	0.07	0.065	-	0.056	-	0.062	0.054	0.059	0.059	0.052
NLPR	*S_m_*↑	0.917	0.925	0.922	0.924	0.918	0.922	0.928	0.923	0.936	0.93	0.932	0.931	0.935	0.941	0.941
	*F_m_*↑	0.912	0.916	0.915	0.922	0.917	0.914	0.926	0.918	0.93	0.929	0.925	0.927	0.943	0.94	0.941
	*E_m_*↑	0.94	0.962	0.949	0.956	0.95	0.95	0.957	0.956	0.961	0.961	0.965	0.954	-	0.968	0.969
	*M*↓	0.03	0.022	0.026	0.024	0.024	0.026	0.021	0.024	0.02	0.021	0.021	A0.024	0.018	0.018	0.017
NJU2K	*S_m_*↑	0.903	0.903	0.915	0.912	0.904	0.914	0.908	0.918	0.926	0.926	0.922	0.922	0.927	0.935	0.931
	*F_m_*↑	0.913	0.903	0.925	0.925	0.916	0.923	0.918	0.927	0.933	0.939	0.923	0.926	0.941	0.943	0.938
	*E_m_*↑	0.925	0.944	0.936	0.94	0.935	0.938	0.937	0.949	0.95	0.954	0.954	0.942	-	0.957	0.958
	*M*↓	0.046	0.043	0.038	0.038	0.039	0.038	0.039	0.033	0.032	0.029	0.032	0.036	0.027	0.027	0.026
STEREO	*S_m_*↑	0.913	0.903	0.892	0.915	0.898	0.91	0.911	0.914	0.918	0.911	0.908	0.913	0.923	0.919	0.919
	*F_m_*↑	0.909	0.903	0.897	0.914	0.91	0.906	0.91	0.908	0.923	0.921	0.908	0.915	0.934	0.926	0.926
	*E_m_*↑	0.93	0.944	0.92	0.938	0.939	0.935	0.938	0.941	0.949	0.946	0.947	0.939	-	0.947	0.951
	*M*↓	0.042	0.043	0.048	0.039	0.039	0.042	0.037	0.035	0.034	0.035	0.038	0.038	0.027	0.033	0.031
DES	*S_m_*↑	0.937	0.929	0.927	0.939	0.917	0.92	0.924	0.94	0.925	0.946	-	0.946	-	0.945	0.947
	*F_m_*↑	0.943	0.919	0.937	0.949	0.929	0.937	0.937	0.944	0.938	0.952	-	0.949	-	0.952	0.956
	*E_m_*↑	0.961	0.968	0.953	0.971	0.955	0.938	0.953	0.974	0.946	0.98	-	0.971	-	0.973	0.98
	*M*↓	0.021	0.022	0.22	0.017	0.023	0.022	0.018	0.015	0.022	0.013	-	0.017	-	0.016	0.014
SIP	*S_m_*↑	0.867	0.879	0.856	0.879	0.861	0.881	0.871	0.892	0.896	0.892	0.894	0.903	0.893	0.911	0.9
	*F_m_*↑	0.889	0.885	0.88	0.906	0.891	0.9	0.895	0.91	0.919	0.919	0.902	0.924	0.922	0.936	0.93
	*E_m_*↑	0.9	0.923	0.888	0.916	0.909	0.918	0.913	0.931	0.931	0.927	0.932	0.935	-	0.944	0.935
	*M*↓	0.063	0.051	0.066	0.053	0.057	0.051	0.052	0.044	0.043	0.043	0.043	0.041	0.041	0.035	0.04

**Table 2:** Effective analysis of the proposed modules on two datasets. The best results are shown in bold.

Models	LFSD				STEREO			
	*M*↓	*S_m_*↑	*F_m_*↑	*E_m_*↑	*M*↓	*S_m_*↑	*F_m_*↑	*E_m_*↑
**Ours**	**0.052**	**0.893**	**0.909**	**0.925**	**0.031**	**0.919**	**0.926**	**0.951**
ResNet50	0.084	0.835	0.864	0.868	0.044	0.893	0.0.9	0.927
w/o EGCIM	0.067	0.87	0.887	0.902	0.035	0.913	0.922	0.946
w/o DEM	0.064	0.875	0.893	0.906	0.032	0.917	0.925	0.949
w/o CIM	0.066	0.869	0.887	0.901	0.033	0.914	0.923	0.947

**Table 3:** Complexity comparison and performance on LFSD and NLPR datasets. The best two results are shown in red and green fonts, respectively.

Backbone	Model	Num_Parameters ↓	FLOPs ↓	LFSD *F_m_* ↑	NLPR *F_m_* ↑
CNN	CMW	85.7 M	208 G	0.899	0.912
	HiDANet	59.8 M	73.6 G	0.877	0.922
	JLDCF	143.5 M	211.1 G	0.862	0.916
	AFNet	242 M	128 G	0.902	0.93
Transformer	SwinNet	198.7 M	124.3 G	0.903	0.94
	**Ours**	175.6 M	96 G	0.909	0.941

## Data Availability

Not applicable.
